# CD13 is a bona-fide marker of bovine pre-adipocytes with potential in cultivated fat applications

**DOI:** 10.1038/s41538-026-00711-z

**Published:** 2026-01-13

**Authors:** Seungmee Lee, Thomas Thrower, Susanna E. Riley, Cristina L. Esteves, F. Xavier Donadeu

**Affiliations:** Division of Translational Bioscience, The Roslin Institute and Royal (Dick) School of Veterinary Studies, University of Edinburgh, Midlothian, UK

**Keywords:** Biological techniques, Biotechnology, Cell biology, Molecular biology, Stem cells

## Abstract

Despite its significant potential, cultivated fat manufacture remains a relatively inefficient process, as it primarily uses source preparations of animal-derived mesenchymal stem/stromal cells (MSC) that are poorly defined and highly heterogenous in nature, containing only relatively minor fractions of bona-fide adipocyte progenitors. The aim of this study was to use RNA-sequencing of clonal MSC populations from cattle to identify cell surface marker(s) of bona-fide pre-adipocytes to be used for efficient enrichment of MSCs, with a view to future cultivated fat applications. Adipose-derived MSC populations (n = 5 animals) were grown clonally from single cells and subsequently tested for adipogenic capacity. Adipogenic (A) and non-adipogenic (N) clones (n = 10/group) thus identified were bulk RNA-sequenced. A total of 35 cluster of differentiation (CD) genes were identified among differentially expressed transcripts, of which CD13, CD141, CD36, CD55 and CD34 were selected for further testing using flow cytometry in bovine MSCs. All antigens except CD13 were detected at negligible levels. FACS was then used to sort MSCs (n = 4 animals) into CD13+ and CD13- fractions. Sorted CD13+ cells were larger and flatter, grew significantly slower, and expressed substantially higher levels of adipogenic regulators (PPARG and CEBPA) compared to CD13- cells. Moreover, on average, adipogenic efficiency was 10.3-fold higher in CD13+ than CD13- cells, as demonstrated by BODIPY staining and confirmed by differential expression of mature adipocyte markers (FABP4, ADIPOQ, LEP), while expression of alternative lineage markers (chondrogenic and osteogenic) and ability to differentiate into bone and cartilage were both similar for CD13+ and CD13- cells. In summary, we identified CD13 as a bona-fide marker of pre-adipocytes in bovine and demonstrated the potential of using CD13-based cell selection for enriching MSC populations for cultivated fat purposes.

## Introduction

Animal fat is a key component of food as it provides characteristic taste and texture as well as essential nutrients and energy. There has recently been increased interest in cell-based manufacture of fat (i.e., without using animals), also known as cultivated fat. This has important potential applications in the rapidly expanding field of cultivated meat as well as wider food sector, e.g., as additive to traditional or plant-base foods^1^. However, at present, production of fat from cells in culture is a relatively inefficient process, preventing scaling-up as required for industrial use.

Primary mesenchymal stem/stromal cell (MSC) cultures are the most common and readily available source of in vitro-produced fat. MSCs are typically obtained by harvesting the stromal vascular fraction (SVF) from adipose tissue samples and growing this on plastic to selectively expand adherent cells. MSC populations thus generated are heterogenous in nature, containing a mixture of different progenitor cell types in addition to terminally differentiated cells, with only a fraction of progenitors able to differentiate into adipocytes^2–4^. Moreover, MSCs have limited ability for self-renewal, further contributing to their finite and variable fat-forming capacity. Importantly, a lack of knowledge on adipocyte progenitor biology has long hindered development of protocols for selective enrichment of such populations from heterogeneous MSC preparations to allow efficient production of fat in vitro.

Single cell sequencing has recently provided unprecedented insight into the heterogeneity of adipose progenitor populations in mice and humans, revealing a hierarchy comprising highly proliferative, multipotent stem cells that give rise to both committed adipocyte progenitors and non-adipogenic cell types^2,5^. The latter include adipogenesis regulators (Aregs) which can inhibit differentiation of neighbouring cells, and structural Wnt-regulated adipose tissue (SWAT) cells which are putatively involved in maintaining the stem cell niche. These populations are defined and can be selectively enriched based on distinct cell surface marker profiles. However, progenitor populations can have distinct characteristics, including in cell surface marker profiles, depending on species (i.e. mice vs. humans) or fat depot of origin, the latter being exemplified by the distinct presence in visceral fat of regulatory immunogenic cells such as fibro-adipogenic progenitors and mesothelial cells^2^.

Substantially less information exists on adipocyte progenitors and their defining molecular markers in livestock compared to model species. Some progress has been facilitated recently by the growing interest in cultured meat. Thus, two studies^6,7^ reported on FACS isolation and subsequent expansion in culture of distinct adipocyte progenitor populations from fresh muscle samples from cattle using different multi-antibody panels (CD29 + /CD56-/CD45-/CD31- and CD26-/CD146 + /CD31-/CD45-, respectively). Another study used a similar antibody panel to isolate an adipogenic fraction (CD29 + /CD140a + /CD45-/CD31-) from porcine subcutaneous fat-derived SVF^8^. Surface marker selection in these studies was guided by information available from species other than cow or pig. Moreover, purification of adipocyte progenitors from fresh tissue samples has several disadvantages that precludes their practical use in cultivated fat applications. On one side, it requires of multi-marker antibody panels to selectively sort relatively rare cell populations in highly heterogeneous samples thus yielding, at a relatively high cost, very small numbers of cells, not suitable for scaling up^6,8^. Further, using fresh tissue extracts such as SVF invariably requires collection of samples from animals, a procedure which, by definition, culture meat seeks to avoid or minimise. Using stocks of MSCs already expanded in culture for this purpose provides a more attractive option, as it reduces the level of heterogeneity in the starting cell population (thus in principle allowing the use of a simpler set of antibodies to isolate cells of interest), it minimises the need for animal sampling, and is more amenable to scaling up.

Thus, the aim of this study was to develop a simplified FACS protocol for selectively and reliably enriching adipogenic progenitor cells from bovine MSC populations in culture. To achieve this, we set to identify suitable surface markers using an unbiased approach through transcriptomic analyses of clonally derived cell populations with differing adipogenicity, followed by FACS analyses of identified targets.

## Results

### Derivation of clonal MSC populations with differing adipogenic capacity

**Fig. 1:**
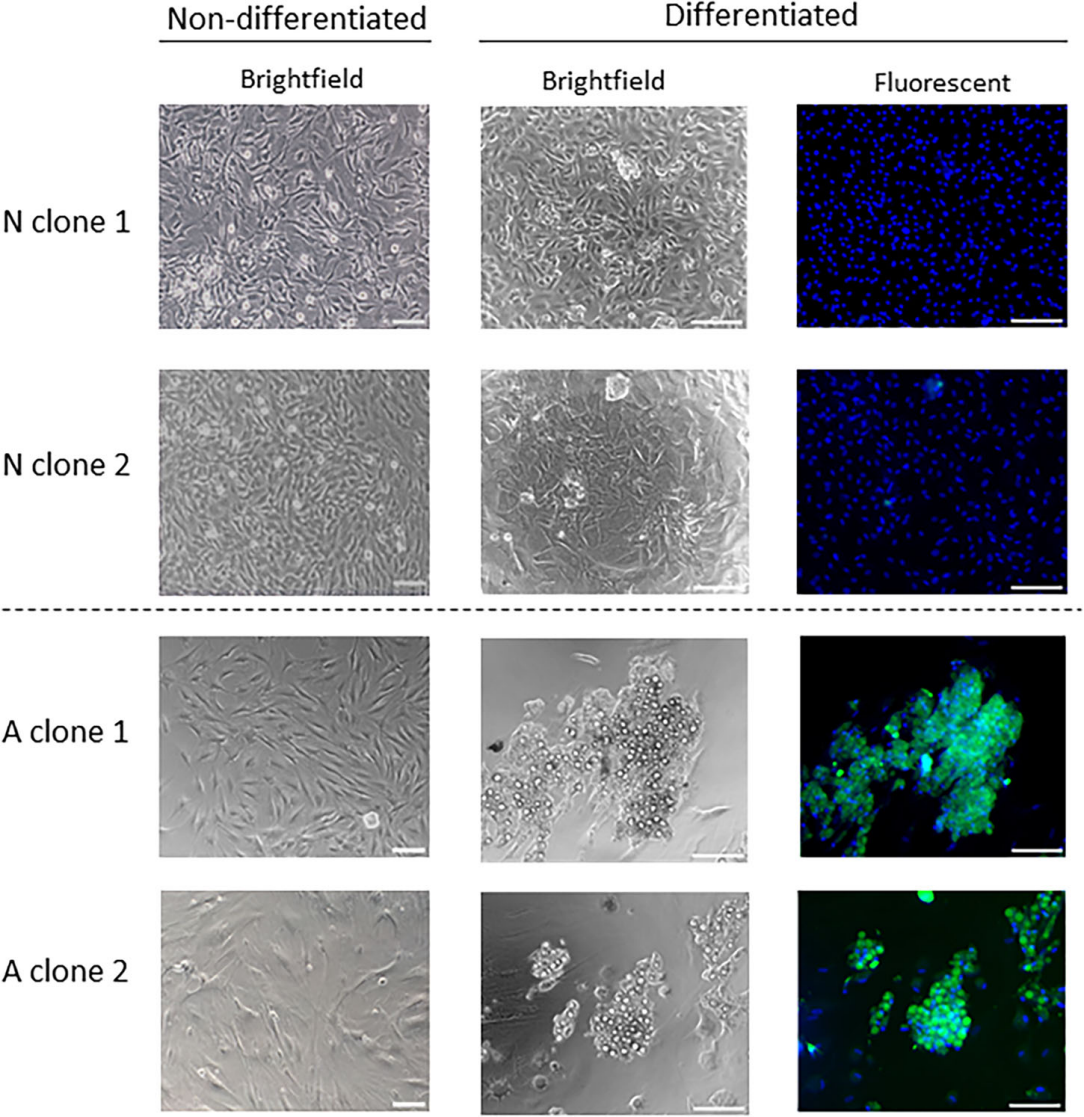
Adipogenic capacity of bovine MSC clones. Microscope images of representative Non-adipogenic (N) and Adipogenic (A) clones before (left column) and after (middle and right columns) differentiation. Bodipy-stained adipocytes in differentiated A clones (in green) are shown in fluorescent images in right colum, where nuclear staining (Hoechst 33342) is shown in blue. Bar = 100 µm.

Cells with characteristics typical of bovine MSCs, including immunophenotype^9,10^, were derived from adipose tissue samples from each of 5 calves (Supplementary Fig. 1), then each sorted into single cells and grown clonally. Less than half of cells seeded (23-47%, depending on animal of origin) yielded clones that reached confluency in 96-well plates (total, 589 clones). These were individually picked at 10 to 17 days after seeding and were each split simultaneously into 96- and 24-well plates. Once they reached confluency, cells in 96-well plates were tested for adipogenic capacity (T1) whereas cells in 24-well plates were expanded further before a second differentiation test (T2). Out of all clones, 109 (19%) were able to produce adipocytes at T1, as indicated by visual inspection and confirmed, for a fraction of clones, by staining with BODIPY (Fig. 1, right column). Of those, 39 clones (7% of total) were able to both expand further and maintain their ability to produce adipocytes at T2. These clones were hereafter referred to as adipogenic (A) whereas clones that failed to differentiate at T1 were referred to as non-adipogenic (N). Retrospectively, N clones grew faster and reached confluency earlier than A clones (11.4 ± 1.1 vs 13.3 ± 1.8 days after single-cell seeding, p = 0.007). In addition, cells in A clones were larger, flatter and looser in appearance than those in N clones (Fig. 1, left column).

### Transcriptome analyses of bovine MSC clonal populations reveal cell surface markers associated with adipogenic potential

**Fig. 2:**
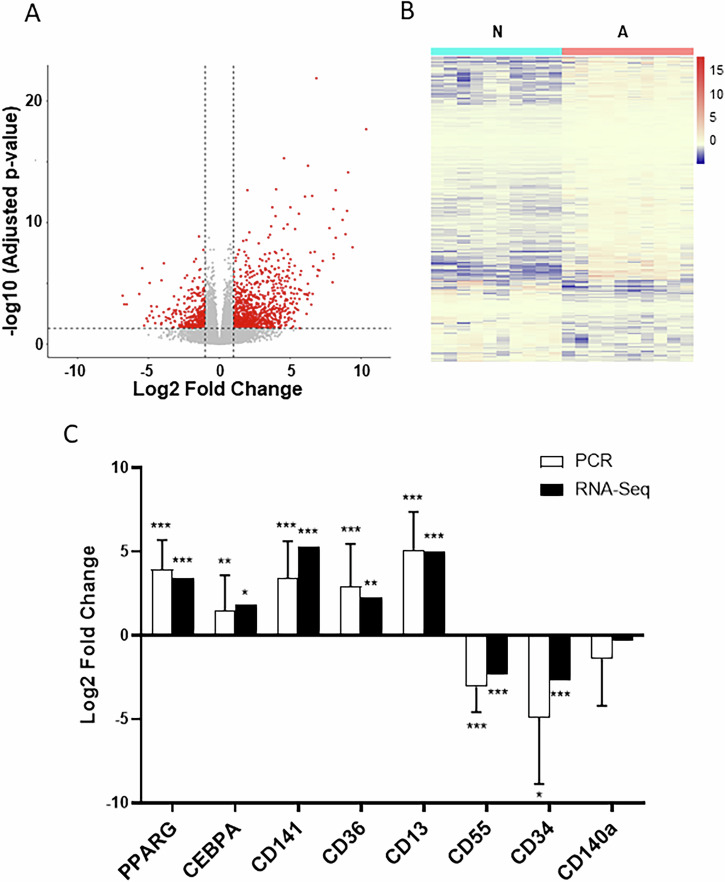
Comparative transcriptome analyses of Non-adipogenic (N) and Adipogenic (A) clones. **A** Volcano plot showing all unique transcripts (represented by dots) obtained by RNA sequencing (n = 10 clones/group). Differentially expressed (DE) genes (*Padj* < 0.05, ≥2-fold) are shown by red dots above/outside dotted-line thresholds. **B** Heat map representing all DE transcripts (*Padj* < 0.05, ≥2-fold). Each row represents a transcript and each column a N or A clone. The colour scale illustrates the relative expression level of each transcript. **C** Comparative log2 fold-change expression values of selected transcripts obtained by RNA-seq (Suppl Table 1) or qPCR (values shown as mean + SD) of N and A clones. QPCR validation was performed on an extended set of clone samples (*n* = 14/group). In all cases, significant up-regulation or down-regulation of transcript levels in A relative to N clones is indicated by ***(*Padj* < 0.001), **(*Padj* < 0.01) or *(*Pad*j < 0.05).

RNA sequencing was used to unbiasedly identify surface markers of adipogenic potential in bovine MSC clones. The ten clones with the overall highest differentiation capacity as indicated by percentage of cells stained with BODIPY after differentiation, were selected for RNA sequencing. These clones originated from MSC populations from four of the five animals. The same number of N clones from each animal were used as controls in a paired design. The two types of clones had clearly distinct transcriptional profiles, with a total of 3344 differentially expressed genes (DEGs) being identified (*Padj* < 0.05), of which 1,305 by ≥2-fold (Fig. 2A, B, Supplementary Table 1); of these, 932 genes were upregulated in A clones including, as expected, numerous positive regulators of adipogenesis such as the master transcriptional regulators, PPARG and CEBPA (Fig. 2C, Supplementary Table 1), and their targets including PLIN1 and AGPAT2, as well as the lipogenic gene, FASN (Supplementary Table 1). Moreover, known genetic inhibitors of adipogenesis, for example CTBP1, RBP1 and RUNX1T1, were among downregulated transcripts (Supplementary Table 1).

**Fig. 3:**
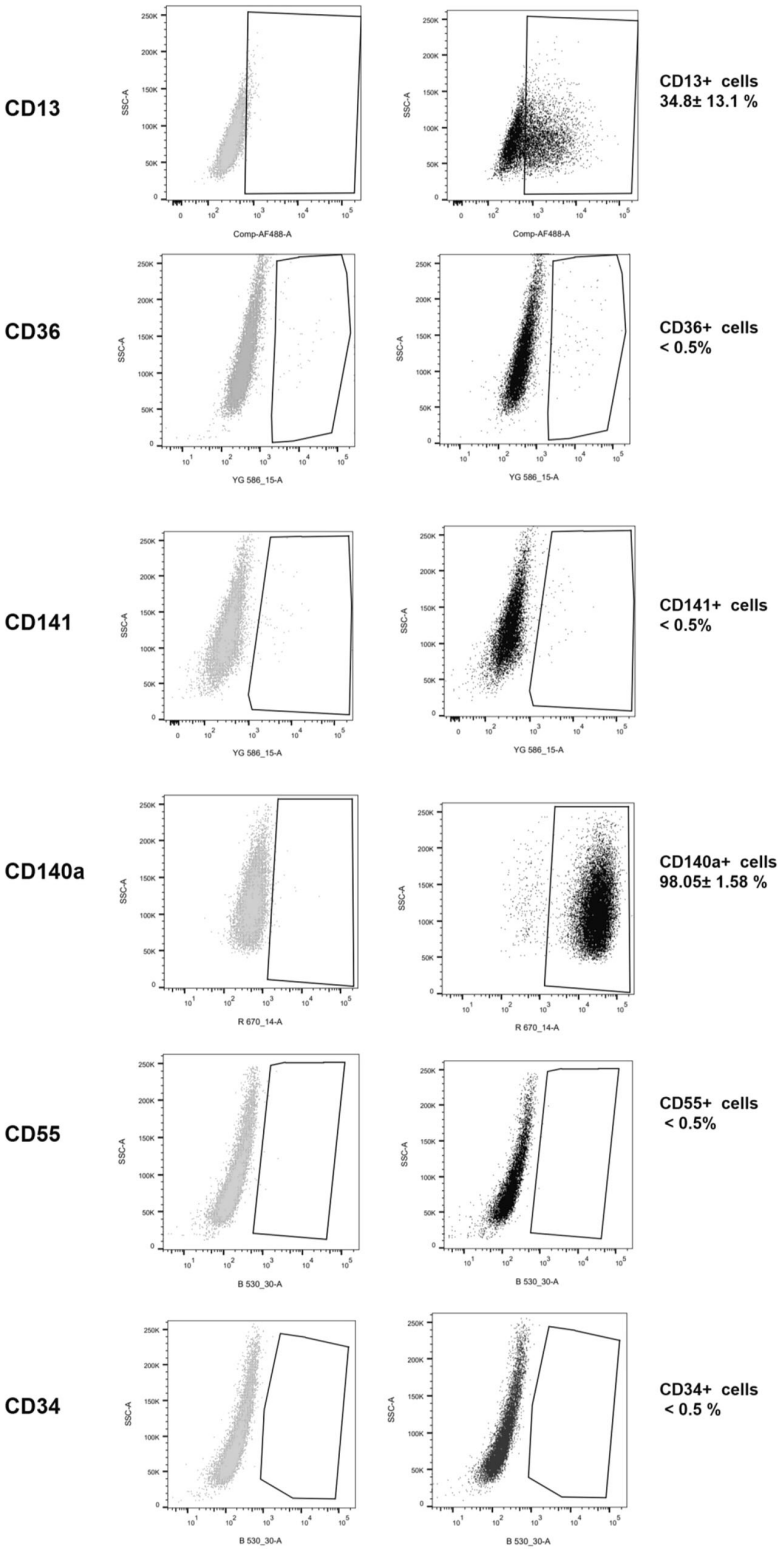
Flow cytometry analyses of surface markers in bovine MSCs. Representative dot-plots from flow cytometry of cells stained with CD13, CD36, CD141, CD140a, CD55 and CD34 (right panel shows % positive cells as mean ± SD), and corresponding isotype controls (left panel). n = 3 to 5 biological replicates from different animals. SSC-A side scatter area.

Importantly, a total of 35 DEGs (*Padj* < 0.05, ≥2-fold change) corresponded to established clusters of differentiation (CD) (Supplementary Table 2), including several reportedly associated with MSCs^2,5^. To identify markers suitable for FACS enrichment of bovine pre-adipocytes, a subset of such CD genes including CD13 (ANPEP), CD141 (THBD), CD36, CD55 and CD34, were selected for further testing. These were genes for which 1) differences in expression between N and A clones were >4-fold and confirmed by QPCR (Fig. 2C), 2) relative transcript abundance in bovine MSCs was at least moderate (in all cases >100 counts on average across all samples, Supplementary Table 2), and 3) commercial antibodies that cross-reacted with cow were available. For completeness, we also included CD140a (PDGFRA), a widely regarded, cross-species pre-adipocyte marker which, although highly expressed in bovine MSCs (see files under Data availability), did not feature among DEGs in our RNA-sequencing data. Antibody testing using flow cytometry revealed a sizeable CD13+ population (20-52%) in MSC preparations (Fig. 3). In contrast, the proportions of cells positive for CD141, CD36, CD55 or CD34 were almost negligible (≤0.5% on average for all markers), whereas almost all cells were positive for CD140a. Based on these results, we selected CD13 for subsequent enrichment of bovine MSC populations using FACS.

### CD13 effectively enriches for adipogenic progenitors in bovine MSC populations

**Fig. 4:**
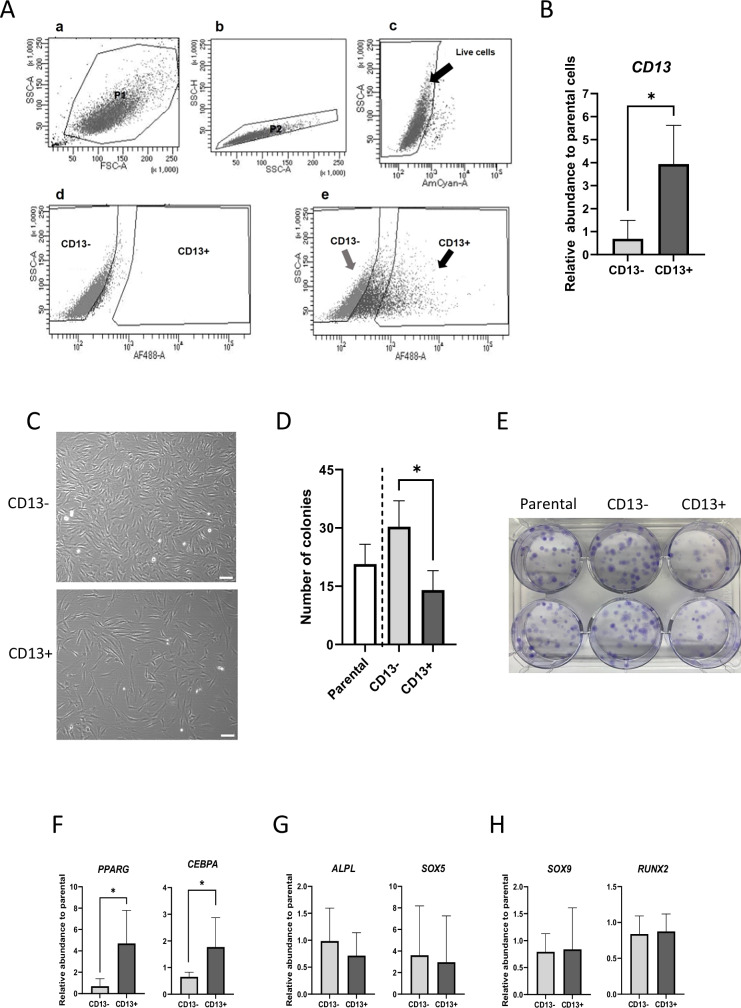
Sorting, culture and characterisation of CD13 cell fractions. **A** Sequence of flow cytometry dot-plots obtained during FAC sorting of MSCs, displaying original sample events (a; FSC-A vs SSC-A), followed by selection of singlets (b; SSC-A vs SSC-H) and live cells (c; zombie aqua-negative), isotype control (d), and CD13 sorted cells (e; grey and black dots correspond to CD13- and CD13+ fractions, repectively). FSC-A forward scatter area, SSC-A side scatter area, SSC-H side scatter height. **B** Relative CD13 transcript levels in CD13- and CD13+ fractions, normalised to levels in parental (unsorted) cells. **C** Representative brightfield images of cultured CD13- and CD13+ cell fractions one week after sorting. Scale bar = 100 µm. **D** Average numbers of colonies obtained from parental, CD13- and CD13+ populations, following single-cell sorting and plating in 96-well plates. **E** Representative images of CFUs following low density plating of parental (unsorted), CD13- and CD13+ populations. **F**, **H** Relative transcript levels of adipogenic (**F**), osteogenic (**G**) and chondrogenic (**H**) markers in CD13- and CD13+ cell fractions, normalised to levels in parental cells. In all panels, quantitative values are shown as mean ± SD (n = 4 biological replicates from different animals/group). *, *P* < 0.05.

CD13+ and - fractions were obtained from MSCs at passages 3 or 4 from each of four animals (Fig. 4A, B). Cell numbers (mean ± SD) obtained for CD13+ and CD13- fractions were 209186 ± 76305 and 386270 ± 184916, respectively, corresponding to 30.15 ± 25.25% and 43.38 ± 17.24% of cells in the parental MSC populations. Sorted cells were expanded separately and were characterised alongside unsorted parental MSCs. Compared to CD13-, CD13+ cells were large and flat in morphology with more cytoplasmic protrusions (Fig. 4C), and grew significantly slower, as indicated by proportionally longer doubling times (28.13 ± 1.03 h vs 25.00 ± 0.92 h, P = 0.04). Consistent with this, colony forming unit (CFU) assays showed reduced clonal growth of CD13+ compared to CD13- cells (Fig. 4D, E).

**Fig. 5:**
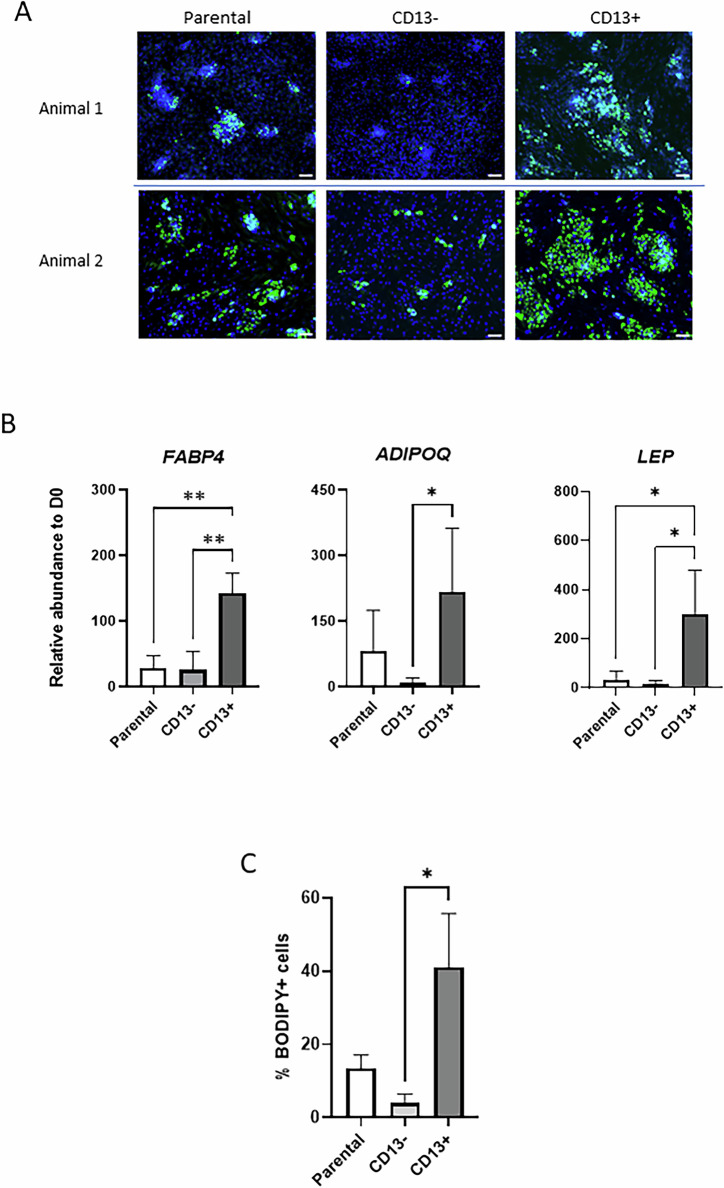
Analyses of adipogenic potential of CD13-sorted cell fractions. **A** Representative fluorescence images of parental, CD13- and CD13+ cell fractions from two different animals following 11 days in adipogenic differentiation media. Lipid (BODIPY, green) and nuclear (Hoechst 33342, blue) staining are shown. Scale bar = 100 µm. **B**, **C** Relative transcript levels of mature adipogenic markers (**B**), and percentage of adipocytes over total cells (**C**, determined by BODIPY staining) in parental, CD13- and CD13+ populations following 11 days in adipogenic differentiation media. All quantitative values are shown as mean ± SD (*n* = 4 biological replicates from different animals/group). *, *P* < 0.05. **, *P* < 0.001.

Moreover, transcriptional profiling showed substantial induction of master adipogenic regulators (PPARG and CEBPA) in CD13+ compared to CD13- cells (Fig. 4F). In contrast, there were no differences in mean levels of alternative lineage regulators, namely, osteogenesis (Fig. 4G) and chondrogenesis (Fig. 4H), between the two cell types. In agreement with these results, CD13+ cells were distinctively adipogenic compared to parental and, especially, CD13- cells, as demonstrated by BODIPY staining (Fig. 5A) and by transcript levels of several mature adipocyte markers in cells after 11 days of differentiation (Fig. 5B). Remarkably, albeit variable among MSC preparations from different animals, adipogenic efficiency (taken as % of cells stained with BODIPY at Day 11 of differentiation) was, on average, 10.3-fold higher in CD13+ than CD13- fractions (Fig. 5C; Supplementary Table 3), with adipocytes making up to 80% of total cells after differentiation in some CD13+ preparations (e.g. Fig. 5A, Animal 2). Moreover, no obvious differences were detected in the ability of CD13+ and CD13- populations to undergo osteogenesis or chondrogenesis (Supplementary Fig. 2).

Finally, to determine whether the CD13 immunophenotype was stably maintained during sequential cell passaging, we analysed CD13+ and CD13- fractions by flow cytometry at passages 3 and 5 following initial sorting. We found that in CD13+ fractions about half of cells remained CD13+ positive at both passages 3 and 5 (mean ± SD, 54.13 ± 37.34% and 50.40 ± 34.65%, respectively), with relatively smaller proportions of CD13+ cells being present in CD13- fractions (20.46 ± 11.78% and 10.67 ± 8.91%, respectively). Values were again highly variable across MSC preparations (Supplementary Fig. 3).

## Discussion

The ability to efficiently produce farm animal fat at scale will be key to the success of cultured meat and its promised contribution to a sustainable food supply. An important limitation at present is the low efficiency with which adipocytes can be generated from animal tissue-derived, highly heterogeneous progenitor cell preparations in culture. Developing approaches to robustly source, grow and differentiate enriched populations of bona-fide pre-adipocytes will require greater understanding of adipocyte progenitors and their defining molecular markers in livestock species. Towards this goal, we used transcriptomics analyses of clonal MSC populations for unbiased identification of markers of committed pre-adipocytes in cattle. We then used this information to develop a simple FACS-based protocol for pre-adipocyte enrichment of bovine MSCs. Using this approach, we identified CD13 as a bona-fide pre-adipocyte marker and demonstrated its potential towards generating cultivated fat from bovine with remarkably high efficiency.

Single-cell cloning has been used to study MSC heterogeneity in model species but, to our knowledge, not in livestock. Clonal derivation efficiency in our study (average, 31%) was higher than that reported using human adipose-derived MSCs, where only 12 clones were successfully expanded from a total of 288 single cells plated^11^. Moreover, the overall frequency of adipogenic clones in bovine MSCs (19%) was lower than those reported for human and mouse MSC clones, which in turn were highly variable^12,13^. The fact that in our study 1) fast-growing clones were selected for, that putatively contained multipotent stem (i.e., non-committed) cell fractions^2^, 2) multiple rounds of expansion were required before enough cells were available for analyses, and 3) adipogenic potential of parental MSC populations was modest (7–24% adipogenic efficiency across populations, Supplementary Table 3), all may have contributed to the relative low frequencies of the adipogenic clones obtained. Nonetheless, our approach yielded sufficient clones with clearly distinct phenotypes (A vs N) to allow for meaningful transcriptome analyses.

Surface cell markers enriched in adipogenic clones included established bona-fide markers of human and mouse pre-adipocytes, such as CD24 and CD36^2,14^. Other such markers were either not enriched, for example CD29 and CD140a, or were downregulated in A compared to N clones, as was the case of CD55 and CD34. Species differences may account for these apparent discrepancies. Moreover, in contrast to the above-cited studies, we used culture-adapted rather than fresh SVF-derived cell populations. Adaptation to culture is associated with distinct immunophenotypic changes in MSCs including abolishment of CD34 and a higher prevalence of CD13^15–18^, consistent with surface marker profiles obtained in the present study. CD13 is a ubiquitous, multifunctional ectopeptidase^19^ that is conservatively expressed in multipotent mesenchymal cells^15,20,21^. Among other functions^22^, CD13 has been reported to regulate adipogenic differentiation of human MSCs^23,24^. Although the mechanism(s) involved have not been elucidated, CD13 may act by inducing adipogenic signalling upstream of PPARG^23^ and, based on its demonstrated involvement in facilitating cholesterol endocytosis in other cell types^19^, could act by promoting lipid uptake by adipose progenitors. Additionally, CD13 may influence pre-adipocyte differentiation through regulatory effects on cell proliferation and cell-to-cell adhesion^19^. Regardless of whether and how CD13 may be causally involved in adipogenic lineage commitment, our results showed that, compared to CD13- cells, CD13+ cells displayed a committed phenotype with loss of stem/progenitor characteristics, as evidenced by differences in cell morphology and clonal growth capacity (Fig. 4C–E), as well as expression of core adipogenic lineage transcripts (Fig. 4F). Moreover, in addition to showing enhanced adipogenic capacity, CD13+ cells displayed similar osteogenic and chondrogenic capacity to CD13- cells, as evidenced by both in vitro differentiation and expression of lineage-specific genes (Fig. 4G, H, Supplementary Fig. 2). This observation is consistent with the now accepted notion that, rather than committing exclusively to one mesenchymal lineage, MSCs become lineage-primed in culture during which they co-express alternate lineage-specific genes, thus maintaining their multipotency [9, 21]. Therefore within an MSC population CD13 may serve as a marker of committed adipocyte progenitors that preserve other lineage capabilities.

Adipogenic capacity can vary considerably with MSC tissue of origin, donor age, as well as passage and differentiation protocol^25–27^. Yet, differentiation efficiencies are rarely reported in MSC literature, particularly in non-model species^27,28^, making comparison of results across studies difficult. Mean adipogenic efficiency of parental unsorted MSC populations in the present study (13.5%) was comparable to that reported recently for bovine subcutaneous-derived MSCs using similar differentiation conditions^27^. Crucially, differentiation efficiency was on average 3.1-fold higher in CD13+ compared to unsorted parental cells (Fig. 5C), demonstrating the significant potential of the sorting approach for enriching bona-fide pre-adipocytes, and providing a simpler alternative to the proposed use of multi-antibody panels for cell enrichment in the context of cultivated meat production^6,7^. Yet, we found differentiation efficiency to be highly variable across cell fractions sourced from different animals (Supplementary Table 3), with CD13+ fractions from only one individual (Animal 2) consistently yielding differentiated cells with an overwhelming majority of adipocytes (82%). Not surprisingly, differentiation efficiency of parental MSCs was highest (24%) in the same animal, thus illustrating the importance of carefully considering animal of origin when sourcing cells for cultivated fat production. In addition, we observed a partial loss of CD13+ phenotype during sequential passaging of sorted cell populations. Loss of CD13+ cells during passaging was variable among cell preparations, being relatively small in two of the three preparations tested (Animals 2 and 3) in which as much as ~70% cells remained CD13+ after 5 passages (Supplementary Fig. 3). Although validation of these results in a larger set of MSC preparations is warranted, this finding demonstrates again the significant potential of our cell enrichment approach for cultivated fat purposes, provided proper consideration is given to the variable effects of animal MSC source. In the end, future considerations on the relative value of using CD13 sorted vs unsorted cells in scaling-up cultivated fat production will need to carefully weigh in the benefits provided by selective cell enrichment against the loss of proliferative capacity during continuous culture, which may impact on net adipocyte production capacity.

An additional point to consider in relation to FACS sorting is that mechanical stress associated with the procedure may conceivably impact on the subsequent differentiation capacity of sorted populations^29,30^, in this case CD13+ cells. In fact, in the context of prospective cultivated fat applications, the use of FACS for enriching progenitor cells of interest may be technically or economically prohibitive. Therefore, studies exploring alternative approaches for CD13+ enrichment of early passage MSCs from livestock species, such as for example magnetic bead-based or even perhaps cell size exclusion, will be warranted in the future. Moreover, since primary MSCs have intrinsically limited proliferative and differentiation capacity, the use of immortalised cell lines will likely be critical for scale-up cultured fat production in the future. In summary, we have identified CD13 as a bona-fide marker of committed adipocyte progenitors in bovine MSCs, and shown the potential of using CD13-based cell enrichment for increasing the efficiency of cultivated fat production.

## Methods

### Derivation and culture of bovine MSCs

All animal procedures were performed with approval from The Roslin Institute (University of Edinburgh) Animal Welfare and Ethical Review Board, and following the UK Animals (Scientific Procedures) Act, 1986. MSCs were derived from adipose tissue samples collected from a total of five Holstein Friesian x Aberdeen Angus calves (1–6 months old) that were euthanized at the University of Edinburgh’s Large Animal Research and Imaging Facility. In brief, after death samples of visceral fat were collected from the inguinal or perirenal region, placed on cold PBS and quickly taken to the laboratory. Samples were then minced and digested in the presence of collagenase II (1 mg/ml; Thermo Fisher Scientific) and BSA (3.5%) for 1 hour at 37 °C with gentle rotation at 100 rpm. Collagenase activity was stopped with DMEM (D5796; Sigma) with 10% FBS (Thermo Fisher Scientific). After removing the lipid layer, the SVF was filtered through a 40 μm cut-off sieve. Cells were cultured initially in DMEM High Glucose supplemented with 10% FBS and 5 ng/ml bFGF (PeproTech, AF-100-18B-1MG) on plastic. Cells were incubated at 39 °C and 5% CO2. After the first passage, cells were grown in plasticware precoated with 0.1% bovine gelatine (Merck, 9000-70-8) in DMEM High Glucose, 10% FBS, 1% Penicillin Streptomycin (Gibco, 15140-122) and 5 ng/ml bFGF. Media was refreshed every 2-3 days. For passaging, cells were washed once in 1xPBS and incubated with 0.25x Trypsin-EDTA (Gibco, 25200-056) for 5–10 min. Trypsin solution was subsequently diluted 1:1 with growth medium and cell pellets were obtained by centrifugation at 300 x g for 5 min. Cells were resuspended in growth medium and either counted with a haemocytometer or directly split 1:4.

Population Doubling Times (PDTs) were calculated from counts of triplicate cell samples as per the Eq. 1 below, with rounding to the nearest quarter hour;

1 $${PDT}=\frac{{Time(h)}\times \mathrm{ln}(2)}{\mathrm{ln}\left(\frac{Final cell number}{Initial cell number}\right)}$$

### Derivation of MSC clones

MSC preparations from each of 5 animals were passaged at least once before single cell sorting. Cells were sorted using BD FACSAria^TM^ fusion, and single cells were seeded on 96 well plates (3 to 5 plates depending on the MSC preparation) that had been pre-coated with 0.1% gelatine. Cells were then cultured in growth media containing 40% conditioned media that had been collected from semi-confluent (60%) autologous MSC cultures and subsequently passed through a 0.45 μm filter^31^. Once confluent, each clone was incubated with TrypLE express and detached cells were split into one well of each of 96- and 24-well plates (2/3 and 1/3 of cells in each clone, respectively). Once they reached confluence, cells in 96-well plates were differentiated to adipocytes as per the protocol below, and cells in 24-well plates were split between a well of a new 96-well plate and a cell aliquot that was saved at –80 °C for later RNA sequencing. Upon reaching confluency, the cells in 96-well plates were differentiated into adipocytes.

### Flow cytometry and FACS

Cells were analysed using a BD Cell Fortessa flow cytometer. In brief, 10^6^ cells were enzymatically harvested using StemPro™ Accutase™ Cell Dissociation Reagent (ThermoFisher Scientific, A1110501) for 5–10 min, then diluted 1:1 with culture medium, and washed twice with PBS before blocking with ice cold FACS buffer (1% BSA + PBS) for 30 min and washing twice with FACS buffer. Samples were then incubated with primary antibody (Supplementary Table 4) for 1 h in the dark at 4 °C, and washed twice with FACS buffer, before a 30 min incubation step with secondary antibody (Supplementary Table 4) at 4 °C in the dark. Samples were washed twice more before staining with live/dead stain (Zombie Violet™ Fixable Viability Kit, BioLegend 423113) and incubation for 15 min at room temperature before washing once more in FACS buffer and resuspension in ice cold FACS buffer. Samples were then run on a BD Fortessa Flow cytometer and data were analysed with FACSDiva software (BD Biosciences, San Jose, CA, USA) or FlowJo (LLC, Ashland, OR, USA). Filters used were 515/30 for Zombie aqua, 525/50 for AF488, 582/15 for YG586/PE and 670/30 for AF647.

For FACS, MSCs were stained for 1 h on ice using primary antibody against CD13 or IgG1 isotype control raised in mouse (Supplementary Table 4) followed by incubation for 30 min on ice with AF488-conjugated secondary antibody. Cells were then sorted on a BD FACSAria Fusion (BD Biosciences, San Jose, USA), and CD13+ and – fractions collected and cultured as described above.

### CFU assays

For CFU assays, 500 and 1000 cells were separately plated in 6-well plates and cultured for 10 days. Colonies were then fixed with paraformaldehyde (PFA, 2%; 30 min), washed with PBS, stained with 1% Crystal violet in 100% Methanol, and counted.

### Cell differentiation assays

*Adipogenesis*. Cells (20,000/cm^2^) were seeded in triplicates onto wells pre-coated with 0.1% gelatine and grown to confluence, after which (Day 0) growth medium was exchanged to standard differentiation media (DMEM High Glucose with 10% FBS, 1% Penicillin-Streptomycin) to which an inducer cocktail was added consisting of 0.5 mM IBMX (Sigma-Aldrich, 41095), 1 µM dexamethasone (Sigma-Aldrich, D4902), 1.8 µM insulin (BioXtra, I9278-5ML), 100 µM Indomethacin (STEMCELL technologies, 73942), 5 µM rosiglitazone^6^ (R2408, Sigma-Aldrich) and 3 µM SB431542^32^ (S4317; Sigma-Aldrich). Media was changed to maintenance medium on Day 4 consisting of standard differentiation media supplemented with 1.8 µM insulin, 5 µM rosiglitazone (R2408, Sigma-Aldrich) and 3 µM SB431542, and refreshed every 48 h up to Day 11.

Differentiated cells were either processed for RT-qPCR as described below, or were fixed in 4% PFA (VWR chemicals, VWR-P38-Sh) and stained with BODIPY™ 493/503 (4 mM, 1:2500, Invitrogen, D3922) and Hoechst 33342 (1 mg/ml, 1:1500, Invitrogen, H1399) for 15 min before imaging. For this, three representative images from triplicate samples/wells were captured using Zeiss Axio Observer Z1 Microscope (Objective: EC Plan-Neofluar 10x/0.30 Ph1 magnification, Zeiss, Germany). Adipocytes were defined as distinct cells containing BODIPY positive lipid droplets, and differentiation efficiency was defined as number of adipocytes/total nuclei. Both adipocytes and nuclei were analysed manually using ZenBlue imaging software (version 3.1).

*Osteogenesis*. Cells were differentiated as previously described^33^. In short, once they reached 90% confluence cells were placed in a mix of high glucose and low glucose DMEM (50:50 v/v; Sigma-Aldrich) supplemented with 10% FBS, 1% Penicillin/Streptomycin, 100 nM dexamethasone (Sigma-Aldrich), 10 mM sodium β-glycerophosphate (Sigma-Aldrich) and 0.1 mM stabilised ascorbic acid (Sigma-Aldrich). After 3 days, cells were switched to DMEM low glucose supplemented as above and cultured for up to 17 days with media change every 3 days. Control cells were maintained in high glucose DMEM, 10% FBS, and 1% Penicillin/Streptomycin. After differentiation, cells were either processed for RT-qPCR or were fixed in PFA (4%) for 15 min and stained with Alizarin Red (2%; pH 4.2) for 30-45 min before imaging in a Zeiss Axiovert 25 Inverted Phase microscope using Zen Blue software (Advanced Micro Devices).

*Chondrogenesis*. Cells were differentiated using the StemPro Chondrogenesis Differentiation Kit (A1007101, Thermofisher). Briefly, cells were seeded in 10 µl of micro-masses in a 96 well plate (80,000 cells/each) and incubated for 2 h in a humidified chamber in the incubator before differentiation medium was added. After 14 days, the chondrogenic micro-masses were either processed for RT-qPCR or were fixed in PFA (4%) for 15 min and stained for 30-45 min with Alcian Blue (1%; Sigma) before imaging in a Zeiss Axiovert 25 Inverted Phase microscope using Zen Blue software (Advanced Micro Devices).

### RNA-sequencing

Cell samples were trypsinised, washed twice with PBS, resuspended in TRIzol reagent (Invitrogen, 15596026), and stored at –80 °C. For RNA extraction, cells homogenised in TRIzol and 1-bromo-3-chloropropane (Sigma-Aldrich, B62404**)** were incubated at room temperature for 3 min then centrifuged for 15 min (12000 x g for 4 °C). Aqueous layers containing RNA were removed and purified manually according to manufacturer’s protocols.

RNA sequencing was performed by Genewiz UK, Azenta Life Sciences (Takeley, Essex, UK). Briefly, the RNA sequencing library was prepared using the NEBNext Ultra II Directional RNA Library Prep Kit following manufacturer’s instructions (New England Biolabs) then validated using DNA Kit on an Agilent 5600 Fragment Analyser (Agilent Technologies) and quantified on a Qubit 4.0 Fluorometer (Invitrogen). The library was then sequenced using the Illumina NovaSeq 6000 platform according to manufacturer’s instructions using a 2×150 bp Paired End configuration (v1.5). NovaSeq Control Software (v1.7) was used to perform image analysis and base calling. Raw sequencing files were converted into fastq files and de-multiplexed using Illumina bcl2fastq (v2.20) software, allowing one mismatch for index sequence identification.

Quality control of the raw data was performed using FastQC, after which adaptor sequences and poor-quality nucleotides were removed using Trimmomatic (v0.36). Trimmed reads were mapped to the reference genome Bos taurus (ARS-UCD1.2.110, ENSEMBL) using the STAR aligner (v2.5.2b). Counts were calculated using the Subread package (v1.5.2) Counts feature, only including unique reads within exons.

Downstream differential expression analysis was performed using DESeq2 software (v1.16.1). Data was analysed, processed, and visualised using standard R coding (v4.3.3) with packages dplyr (v1.1.4), ggplot2 (v3.5.0), ggrepel (v0.9.5), ggthemes (v5.1.0), pheatmap (v1.0.12), RColorBrewer (v1.1-3), rlog (v0.1.0), and tidyverse (v2.0.0). Data were further analysed with QIAGEN IPA software (QIAGEN Inc., https://digitalinsights.qiagen.com/IPA), using DEGs with absolute fold change ≥ 2 and *Padj* < 0.05.

### RT-qPCR

RNA extracted as described above was used for cDNA synthesis. RNA (300 ng) was incubated with 0.5 µl random primers (250 ng/µl, Promega, 1181), 1 µl dNTPs (10 mM, Invitrogen, 18427-013) and nuclease free-water in a thermocycler (Biometra) at 65 °C for 5 min, followed by 5 min at 4 °C. Then, 1 µl DTT (0.1 M, Invitrogen, 18080-93), 1 µl RNasin Plus RNase inhibitor (40 U/µL, Promega, N2611), 1 µl SuperScript III reverse transcriptase (Invitrogen, 18080-93), and 4 µl first strand buffer (5X, Invitrogen, 18080-93) were added and samples incubated at 25 °C for 5 min, followed by 50 °C for 60 min, 70 °C for 15 min and a cooling step at 4 °C for 5 min. No-template- and a no-RT-controls were included. cDNA was kept frozen at –20 °C.

Duplicate cDNA samples (2 µl, 1/40 dilution) were combined with 5 µl SensiFAST SYBR green (Kit, Meridian Bioscience, BIO94020), forward and reverse primer (10 µM, 0.4 µl each, Supplementary Table 5), and 2.2 µl nuclease-free water, and subjected to 95 °C x 2 min, followed by 40 cycles of 95 °C x 5 s, 60 °C x 11 s and 72 °C x 5 s, and a final incubation at 95 °C x 1 min, 60 °C x 30 s and 95 °C x 30 s in a AriaMx Real-time PCR System (Agilent). Target gene copy numbers were calculated using a standard curve made up of 4-fold dilutions of a pool of cDNA, and normalised to the expression of the housekeeping genes, TOP2B and RPL4, within each sample. Data analysis was performed using AriaMx software.

### Statistics

Graphing and statistical analysis were performed in GraphPad 10 or R (v.4.3.3). Data normality was confirmed using a Shapiro-Wilk test. Data were analysed by one-way or two-way ANOVA followed by Tukey’s multiple comparison test, or unpaired t-test when only two means were being compared. Non-normally distributed data were analysed with Kruskal-Wallis test followed by Dunn’s multiple comparison test. Mean and SD are shown for all data.

## Supplementary information

Supplementary figures

Supplementary Table1

Supplementary Table2

Supplementary Table3

Supplementary Table4

Supplementary Table5

## Data Availability

Raw fastq files and counts from RNA sequencing were deposited in NCBI’s GEO under Series GSE281680. All other data is available within the manuscript.
