# Supplementary material for: CD13 is a bona-fide marker of bovine pre-adipocytes with potential in cultivated fat applications
Source: NPJ Sci Food. 2026 Jan 13;10:61. doi: 10.1038/s41538-026-00711-z (PMC12901004; doi:10.1038/s41538-026-00711-z)
Supplement: Supplementary file 1 — Supplementary figures [file 41538_2026_711_MOESM1_ESM.pdf]

A

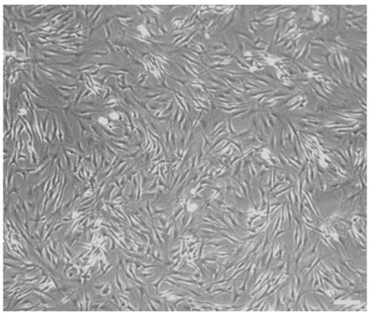

B

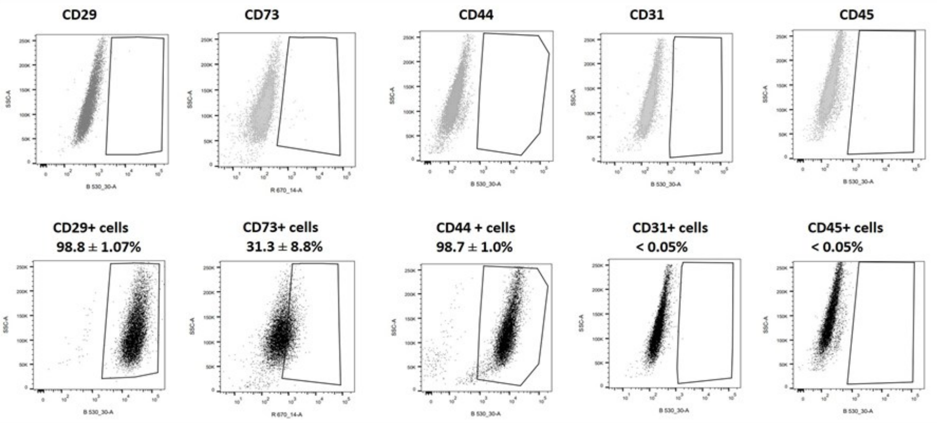

C

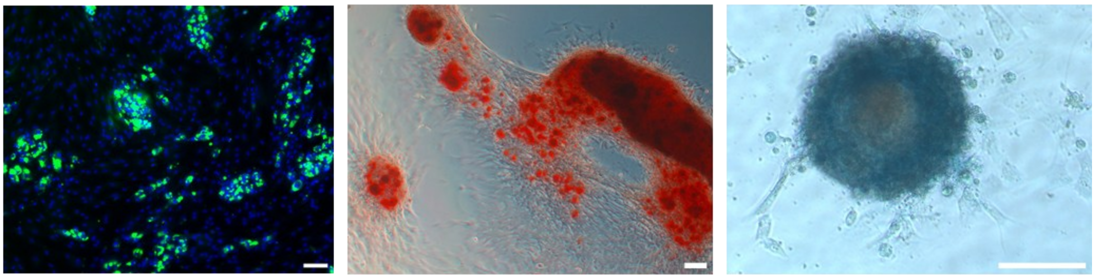

Supplementary Figure 1. Bovine adipose-derived cell populations used in the study had typical MSC features including A) morphology, as illustrated by representative brighfield image, B) expression of cell surface markers, as illustrated by representative flow cytometry dot-plots from cells stained with CD29, CD73, CD44, CD31 and CD45 (lower panel in B, with % positive cells (mean ± SD) indicated; upper panels show isotype controls; *SSC-A* = side scatter area), and C) trilineage differentiation capacity, as shown by images of cells stained with BODIPY (green, left panel), Alizarin red (middle panel) and Alcian blue (right panel), corresponding to adipogenic, osteogenic and chondrogenic lineages. Scale bars = 100 µm.

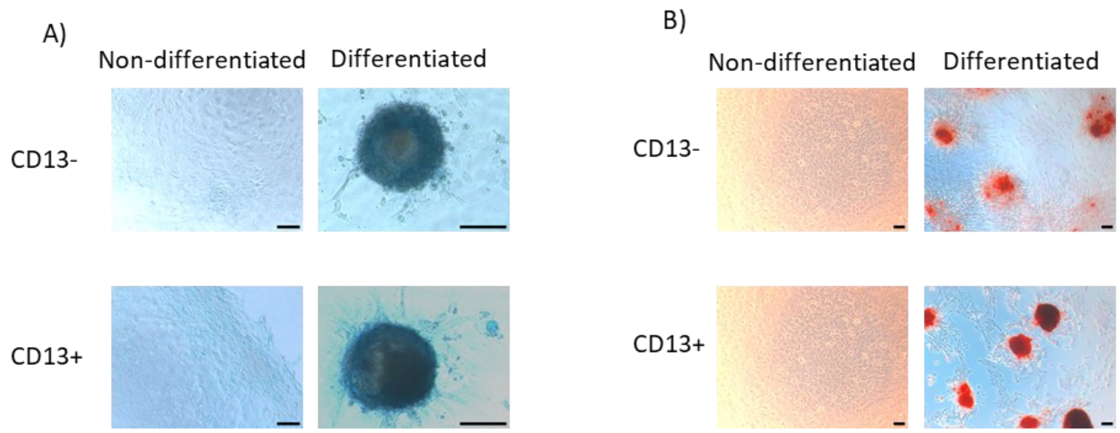

Supplementary Figure 2. Representative images of sorted CD13- and CD13+ cell fractions after differentiation into condrogenic (A, stained with Alcian blue) and osteogenic (B, stained with Alizarin red) lineages. Scale bars = 100 µm.

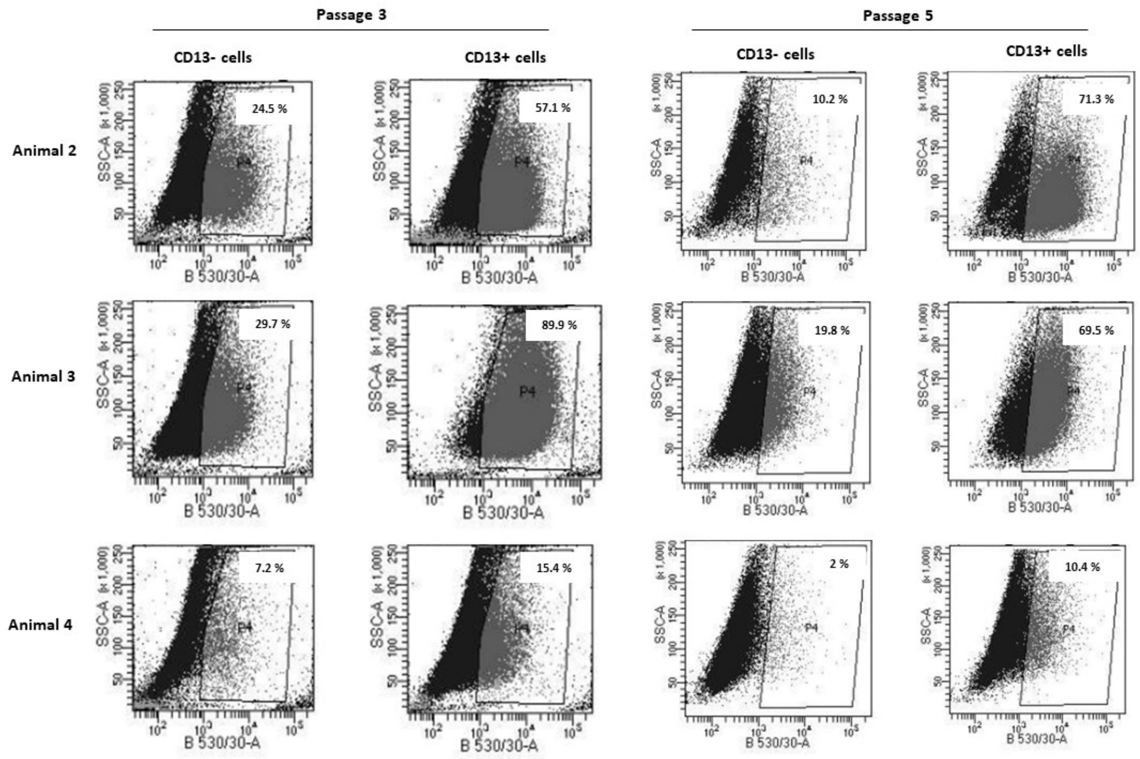

Supplementary Figure 3. Representative dot-plots obtained from flow cytometry of CD13+ and CD13- cell populations stained with CD13 at passages 3 and 5 following initial sorting of MSC preparations from 3 different animals. Top right inset in each panel shows % positive cells. *SSC-A* = side scatter area.
